# Enzymatic activity of *Lactobacillus reuteri* grown in a sweet potato based medium with the addition of metal ions

**DOI:** 10.1186/2193-1801-2-465

**Published:** 2013-09-16

**Authors:** Saeed A Hayek, Aboghasem Shahbazi, Mulumebet Worku, Salam A Ibrahim

**Affiliations:** Food Microbiology and Biotechnology Laboratory, North Carolina Agricultural and Technical State University, 163 Carver Hall, Greensboro, Greensboro, NC 27411 USA

**Keywords:** *L. reuteri*, Metal ions, Mg^2+^, Mn^2+^, *α*–glucosidase, *β*-glucosidases, Acid phosphatase, Phytase

## Abstract

The effect of metal ions on the enzymatic activity of *Lactobacillus reuteri* was studied. The enzymatic activity was determined spectrophotometrically using the corresponding substrate. In the control group, *L. reuteri* MF14-C, MM2-3, SD2112, and DSM20016 produced the highest *α*-glucosidase (40.06 ± 2.80 Glu U/mL), *β*-glucosidase (17.82 ± 1.45 Glu U/mL), acid phosphatase (20.55 ± 0.74 Ph U/mL), and phytase (0.90 ± 0.05 Ph U/mL) respectively. The addition of Mg^2+^ and Mn^2+^ led to enhance *α*-glucosidase produced by *L. reuteri* MM2-3 by 113.6% and 100.6% respectively. *α*-Glucosidase produced by MF14-C and CF2-7F was decrease in the presence of K^+^ by 65.8 and 69.4% respectively. *β*-Glucosidase activity of MM7 and SD2112 increased in the presence of Ca^2+^ (by 121.8 and 129.8%) and Fe^2+^ (by 143.9 and 126.7%) respectively. Acid phosphatase produced by *L. reuteri* CF2-7F and MM2-3 was enhanced in the presence of Mg^2+^, Ca^2+^ or Mn^2+^ by (94.7, 43.2, and 70.1%) and (63.1, 67.8, and 45.6%) respectively. On the other hand, Fe^2+^, K^+^, and Na^+^ caused only slight increase or decrease in acid phosphatase activity. Phytase produced by *L. reuteri* MM2-3 was increase in the presence of Mg^2+^ and Mn^2+^ by 51.0 and 74.5% respectively. Ca^2+^ enhanced phytase activity of MM2-3 and DSM20016 by 27.5 and 28.9% respectively. The addition of Na^+^ or Fe^2+^ decreased phytase activity of *L. reuteri*. On average, Mg^2+^ and Mn^2+^ followed by Ca^2+^ led to the highest enhancement of the tested enzymes. However, the effect of each metal ion on the enzymatic activity of *L. reuteri* was found to be a strain dependent. Therefore, a maximized level of a target enzyme could be achieved by selecting a combination of specific strain and specific metal ion.

## Introduction

Species of the genus lactobacilli are commonly found in a diversity of ecosystems including human, animal, plants, and soil (Barrangou et al. 2011; Song et al. 2012). *Lactobacillus* has been employed in many applications with regard to food, feed, and fertilizers. This genus of lactic acid bacteria is the lead of food fermentation and probiotic applications (Rodríguez et al. 2009; Song et al. 2012). *Lactobacillus* produces several functional enzymes that could help in the digestibility of complex carbohydrates such as indigestible fibers and benefit the human health (Mahajan et al. 2010; Palacios et al. 2007; Raghavendra and Halami 2009; Zotta et al. 2007). For example, *α*–glucosidase (*α*-D-glucoside glucohydrolase, EC 3.2.1.20) is responsible for hydrolyzing glycosidic bonds in oligosaccharides (starch, disaccharides, and glycogen) and releasing *α*-glucose (Krasikov et al. 2001). Deficiency of *α*–glucosidase in human could cause glycogen storage disease II which also known as Pompe (Krasikov et al. 2001). *β*-Glucosidase (*β*-D-glucoside glucohydrolase, EC 3.2.1.21) hydrolyzes all four *β*-linked glucose dimmers in cellulose to produce glucose monomers (Sestelo et al. 2004). Cellulose is considered the highest proportion of plants, and can be hydrolyzed by *β*-glucosidases for both industry and human health. Humans are unable to digest cellulose due to the low levels of cellulases in the gut. *β*-Glucosidase is also used in the production of fuel ethanol from cellulose and in food fermentation to release the aromatic compounds (Sestelo et al. 2004). Acid phosphatase (orthophosphoric monoester phosphohydrolase, EC. 3.1.3.2) and phytase (*myo*-inositol hexakisphosphate 6-phosphohydrolases; EC 3.1.3.26) hydrolyze phytate and reduce its antinutritional properties (Iqbal et al. 1994; López-González et al. 2008; Palacios et al. 2005). The specificity of acid phosphatase and phytase can partially overlapped since acid phosphatase produced by microorganisms has phytase activity (Simon and Igbasan 2002). Phytate is a common fiber that found in cereals, legumes, and nuts, and acts as an antinutrient binding with proteins, lipids, carbohydrates, and metal ions (zinc, iron, calcium, and magnesium). Phytate degrading activity in humans is relatively low (mainly in the small intestine) (Iqbal et al. 1994), so other sources of phytate degrading enzymes are required. Microbial sources of such functional enzymes could be the most promising sources for human health.

Utilization of indigestible fibers and oligosaccharides, not digestible by human enzymes, has been recognized as an important attribute of probiotics (Alazzeh et al. 2009; Gyawali and Ibrahim 2012; Song et al. 2012). Species of *Lactobacillus* that produce functional enzymes such as *α*-glucosidase, *β*-glucosidase, acid phosphatase, and phytase could have an important impact on human health. However, the production capacity of such hydrolyzing enzymes by *Lactobacillus* is strain specific (Bury et al. 2001; Ibrahim et al. 2010; Palacios et al. 2007; Zotta et al. 2009; Zotta et al. 2007). *Lactobacillus reuteri* is known to inhabit the gastrointestinal tract of humans and animals (Casas and Dobrogosz 2000). *L. reuteri* is a special probiotic species since the entire species has been shown to exhibit efficient probiotic functionality (Casas and Dobrogosz 2000) and to produce different functional enzymes (Alazzeh et al. 2009). *L. reuteri* exhibit high activity of *α*-galactosidase and *β*-galactosidase (Ibrahim et al. 2010). Strains of *L. reuteri* have high activity of *α*-glucosidase (Kralj et al. 2005) and *β*-glucosidase (Otieno et al. 2005). These strains also showed the highest phytate degrading activity producing both phytase and acid phosphatase compare to other *Lactobacillus* spp. (Palacios et al. 2007). We have previously shown that *L. reuteri* produce higher *α* spp. (Hayek -glucosidase, acid phosphatase, and phytase than other *Lactobacillus*^2013^).

 In addition to human health applications, these probiotic strains can be also used in animals and plants. However, the enzymatic activity of *Lactobacillus* can be affected by their nutritional requirements such as vitamins metal ions, sugars, and protein (Alazzeh et al. 2009; Hayek and Ibrahim 2013; Ibrahim et al. 2010; Mahajan et al. 2010; Palacios et al. 2005). Nevertheless, even though the nutritional requirements of *Lactobacillus* have been established, controlling, optimizing, and maximizing the enzymatic activity of *Lactobacillus* have many limitations and challenges (Hayek and Ibrahim 2013). Metal ions have been reported in several studies to enhance the enzymatic activity of *Lactobacillus* (Aqel spp. including *L. reuteri*^2012^; Ibrahim et al. 2010; Ozimek et al. 2005; Palacios et al. 2005). For example, the addition of 10 mM of Mn^2+^ caused a significant enhancement in *β*-glucosidase activity while 10 mM of Zn^2+^ or Cu^2+^ resulted in a reduction of *β*-glucosidase of up to 90%. (Jeng et al. 2011). Acid phosphatase was enhanced by Ca^2+^ and Mg^2+^ with a greater effect on Ca^2+^ (Tham et al. 2010). Thus, developing a means to enhance the enzymatic activity of *L. reuteri* may help to solve different digestive problems.

Sweet potatoes (*Ipomoea batatas* (L.) Lam.) (Batatas an Arawak name) are an abundant agricultural product that play a major role in the food industry and human nutrition. Sweet potatoes are a rich source of carbohydrates (mainly starch and sugars), some amino acids, vitamins (vitamin A, vitamin C, thiamin (B1), riboflavin (B2), niacin, and vitamin E), minerals (calcium, iron, magnesium, phosphorus, potassium, sodium, and zinc), and dietary fiber (Broihier 2006; Padmaja 2009). Sweet potatoes also contain other minor nutrients such as antioxidants, triglycerides, linoleic acid, and palmitic acid (Broihier 2006; Padmaja 2009). Previous studies have shown that plant components can support the growth and functionality of probiotic bacteria (Gyawali and Ibrahim 2012). We have previously showed that sweet potatoes could be used to form an alternative low cost medium for the growth of *Lactobacillus* strains (Hayek et al. 2013). *Lactobacillus* strains grown in a sweet potato base medium were also found to produce higher *β*-glucosidase, acid phosphatase, and phytase activities and lower *α*–glucosidase than that in MRS (Hayek 2013). However, the suitability of the sweet potato base medium to study the effect of metal ions on the enzymatic activity of *L. reuteri* was not investigated. Therefore, the objective of this work was to study the effect of metal ions on *α*-glucosidase, *β*-glucosidase, acid phosphatase, and phytase activity of *L. reuteri* growing in a sweet potato based medium.

## Materials and methods

### Media preparation

Sweet potato medium (SPM) was previously developed to support the growth of *Lactobacillus* (Hayek et al. 2013). Fresh sweet potatoes (Covington cultivar) (obtained from Burch Farms in Faison NC, USA) were baked in a conventional oven at 400°C for 1 h. The sweet potatoes were then peeled and blended in a kitchen blender with deionized distilled water (DDW) at a ratio of 1:2. The solution was centrifuged at 7800 *×* g for 10 min using Sorvall RC 6 Plus Centrifuge (Thermo Scientific Co., Asheville, NC, USA) and the supernatant was collected. SPM was then formed by mixing 1 L of supernatant with the following ingredients: sodium acetate (5 g), potassium monophosphate (2 g), disodium phosphate (2 g), ammo-nium citrate (2 g), Tween 80 (1 mL), beef extract (Neogen Corporation, Lansing, MI, USA) (4 g), yeast extract (Neogen Corporation) (4 g), proteose peptone #3 (4 g), and L-Cysteine (1 g). SPM was sterilized at 121°C for 15 min, cooled down, and stored at 4°C then used within 3 days. All ingredients were obtained from Thermo Scientific Co. (Asheville, NC, USA) unless otherwise noted.

### Bacterial culture activation and preparation

*L. reuteri* strains (Table 1) were provided by BioGaia (Raleigh, NC) and stored in the stock collection of the Food Microbiology and Biotechnology Laboratory, North Carolina A&T State University. The strains were activated in SPM by transferring 100 μL of stock culture to 10 mL SPM broth, incubated at 37°C for 24 h, and stored at 4°C. Prior to each experimental replication, bacterial strains were streaked on SPM agar and incubated for 48 h at 37°C. One isolated colony was then transferred to 10 mL SPM broth and incubated at 37°C for next day use.

**Table 1:** ***Lactobacillus reuteri*strains and sources**

***L. reuteri***	Source
MF14-C	Mother fecal isolate
CF2-7F	Child fecal isolate
DSM20016	Mother’s milk
SD2112	Mother’s milk
MM7	Mother’s milk
MM2-3	Mother’s milk

### Culturing with metal ions

Samples of SPM with metal ions were prepared by dissolving 10 mM of either FeSO_4_.4H_2_O, MgSO_4_.7H_2_O, K_2_SO_4_, or Na_2_SO_4_, or 5 mM of either MnSO4._4_H_2_O or CaSO_4_.7H_2_O into batches of 60 mL non-sterile pre-prepared SPM. The use of 10 mM or less of metal ions was established to avoid the hypertonic pressure on bacterial cells (Ibrahim et al. 2010). The used 5 mM of MnSO4._4_H_2_O and CaSO_4_.7H_2_O was required since higher concentrations did not dissolve completely in SPM. Batches of 60 mL SPM without metal ions served as control. Samples were sterilized at 121°C for 15 min, cooled down to room temperature, then inoculated with 3% v/v precultured *L. reuteri* and incubated at 37°C for 16 h. Bacterial growth was monitored by measuring the turbidity (optical density (OD) at 610 nm) at 2 h intervals using a 96-well microplate reader (BioTek Institute, Winooski, VT). At the end of incubation, cultures were divided into two portions of 30 mL each. One portion was used for *α*-glucosidase and *β*-glucosidase determination and the other portion was used for acid phosphatase and phytase determination.

### Enzyme samples preparation

Samples used for *α*-glucosidase and *β*-glucosidase determination were centrifuged at 7800 × g for 10 min at 4°C using Sorvall RC 6 Plus Centrifuge to harvest the bacterial cells. The cells were washed twice with 0.5 M sodium phosphate buffer (pH 6.0) and suspended in 1 mL of the same buffer. Suspended cells were maintained in Eppendorf tubes containing 0.1 mm glass beads and treated with a mini-Beadbeater-8 (Biospec Products, Bartlesville, OK, USA) for a total of 3 min to disrupt the cells. During cells disruption, samples were allowed to rest after each minute for 15 s in an ice bath to avoid overheating. Samples were then centrifuged at 12,000 × g for 20 min using Microcentrifuge 5415 R (Eppendorf, Hamburg, Germany) and supernatant was used for enzyme assay analysis of *α*-glucosidase. Disrupted cells were suspended in a minimum amount of sodium phosphate buffer and used for enzyme assay analysis of *β*-glucosidase.

Samples used for acid phosphates and phytase determination were centrifuged at 7800 × g for 10 min at 4°C to harvest the bacterial cells. The cells were washed with 50 mM Tris–HCl (pH 6.5) and suspended in 1 mL 50 mM sodium acetate-acetic acid (pH 5.5). Suspended cells were disrupted then centrifuged using same procedure as that of samples used for *α*-glucosidase and *β*-glucosidase. Supernatants were used for enzyme assay analysis of acid phosphatase and phytase.

### Determination of *α*-glucosidase and *β*-glucosidase

*α*-Glucosidase and *β*-glucosidase were determined by monitoring the rate of hydrolysis of *ρ*-nitrophenyl-*α*-D-glucopyranoside (*α*-PNPG) and *ρ*-nitrophenyl-*β*-D-glucopyranoside (*β*-PNPG) respectively according to Mahajan and others with some modifications (Mahajan et al. 2010). In this procedure 1 mL of 10 mM of either (*α*-PNPG) or (*β*-PNPG) was mixed with 0.5 mL of the corresponding enzyme sample. Samples were then incubated at 37°C for 20 min. All reactions were stopped by adding 2.5 mL of 0.5 M Na_2_CO_3_. The released yellow *ρ*-nitrophenol was determined by measuring the OD at 420 nm. One unit of *α*-glucosidase or *β*-glucosidase (Glu U/mL) was defined as 1.0 μM of *ρ*-nitrophenol liberated per minute under assay conditions.

### Determination of acid phosphatase and phytase

Acid phosphatase (E.C.3.1.3.2.) was determined by monitoring the rate of hydrolysis of *ρ*-nitrophenyl phosphate (PNPP), and phytase activity was determined by measuring the amount of liberated inorganic phosphate from sodium phytate (Haros et al. 2008). For acid phosphatase, 250 μL of 0.1 M sodium acetate buffer (pH 5.5) containing 5 mM PNPP was mixed with 250 μL of enzyme sample. Samples were then incubated at 50°C for 30 min in a water bath, the reaction was stopped by adding 0.5 mL of 1.0 M NaOH and the released *ρ*-nitrophenol was measured at 420 nm. For phytase, 400 μL of 0.1 M sodium acetate (pH 5.5) containing 1.2 mM sodium phytate was mixed with 250 μL of enzyme sample. Samples were then incubated for 30 min at 50°C in a water bath, the reaction was stopped by adding 100 μL of 20% trichloroacetic acid solution. An aliquot was analyzed to determine the liberated inorganic phosphate (Pi) by the ammonium molybdate method, OD at 420 nm (Tanner and Barnett 1986). One unit of acid phosphatase or phytase (Ph U/mL) was defined as 1.0 μM of *ρ*-nitrophenol or 1.0 μM of Pi liberated per minute under assay conditions.

### Statistical analysis

Each experimental test was conducted three times in randomized block design to evaluate the effect of metal ions on the enzymatic activity of *L. reuteri* in SPM. Mean values and standard deviations were calculated from the triplicate tested samples. R Project for Statistical Computing version R-2.15.2 (http://www.r-project.org) was used to determine significance of differences in the effect of metal ions on the enzymatic activity of the tested *L. reuteri* strains and significance of differences in the enzymatic activity among strains using one way and multi-way ANOVA (analysis of variance) with a significance level of *p* < 0.05.

## Results and discussions

### Effect of metal ions on the growth of *Lactobacillus reuteri*

**Figure 1:**
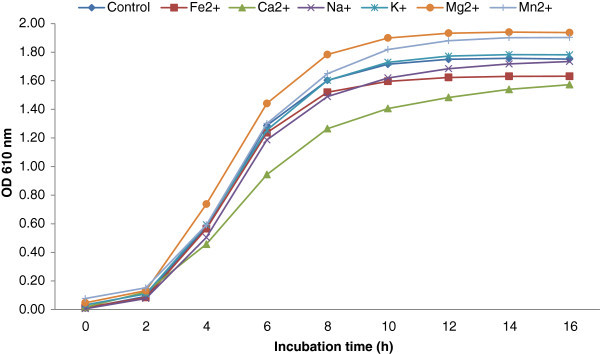
**Effect of adding metal ions on the growth pattern of*L. reuteri*(results of six strains of*L. reuteri*were pooled for each metal ion).**

**Figure 2:**
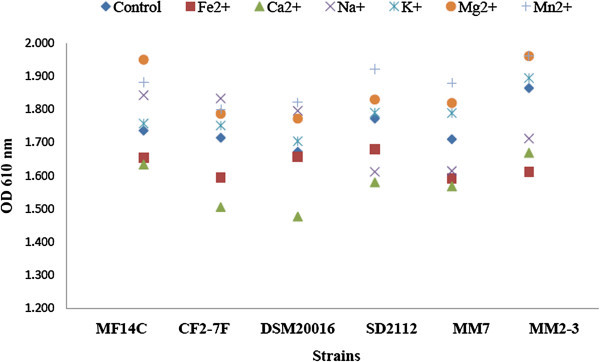
**Effect of adding metal ions on the growth of*L. reuteri*strains after 16 h of incubation.**

The growth of *L. reuteri* was monitored using OD at 610 nm. Figure 1 shows average growth rates of *L. reuteri* strains growing in SPM with added metal ions during 16 h of incubation at 37°C. In the control samples, strains of *L. reuteri* continued to grow and reached an average of 1.75 OD (610 nm) within 16 h of incubation at 37°C. The addition of Mg^2+^ or Mn^2+^ to SPM enhanced the growth of *L. reuteri* to reach an average of 1.94 and 1.90 OD (610 nm) respectively. Fe^2+^ and Ca^2+^ slows down the growth of *L. reuteri* to reach an average of 1.63 and 1.57 OD (610 nm) respectively. Similar growth curves were shown for the strains of *L. reuteri* in the presence of Na^+^ and K^+^ as control group. All tested strains of *L. reuteri* grew better in SPM with added Mn^2+^ or Mg^2+^, and they grew slower in SPM with added Fe^2+^ and Ca^2+^ compared to their in control (Figure 2). The addition of Na^+^ to SPM enhanced the growth of MF14-C, CF2-7F, and DSM20016 but slowed down the growth of SD2112, MM7, and MM2-3. K^+^ showed only slight effect on the growth of the tested strains.

The enhancement *L. reuteri* growth by Mn^2+^ and Mg^2+^ can be explained by that these metal ions are essential for the growth of *Lactobacillus* (Boyaval 1989; Letort and Juillard 2001; Wegkamp et al. 2010). Mn^2+^ helps the cell to deal with reactive oxygen species and serves as an alternative for the absence of a gene encoding a superoxide dismutase (Wegkamp et al. 2010). Mg^2+^ was earlier found to stimulates the growth of *Lactobacillus* and improve its survival (Amouzou et al. 1985). It was shown that Mg^2+^ is the only essential oligoelement for the growth of *Lactobacillus delbrueckii* subsp. *lactis* (Hébert et al. 2004). Mg^2+^ and Mn^2+^ were found to be essential minerals for the growth of *L. plantarum* (Wegkamp et al. 2010). In this experiment we are reporting the enhancement of *L. reuteri* growth by Mn^2+^ and Mg^2+^.

### Induction of *α-*glucosidase by metal ions

In control group, *α*-glucosidase activity produced by *L. reuteri* ranged between 20.65 ± 1.70 and 40.06 ± 2.80 Glu U/mL for MM2-3 and MF14-C respectively (Table 2). In the presence of Mg^2+^, *α*-glucosidase produced by *L. reuteri* MM2-3 was increased by 23.46 units to reach 44.11 ± 3.20 Glu U/mL. *L. reuteri* MF14-C grown in the presence of Mg^2+^ showed the highest *α*-glucosidase activity (61.74 ± 3.09 Glu U/mL) compared to other strains. The addition of Mn^2+^ also enhanced *α*-glucosidase activity *L. reuteri* MM2-3 and MF14-C to reach 41.42 ± 3.66 and 58.31 ± 2.88 respectively. Mn^2+^ enhanced *α*-glucosidase activity for all *L. reuteri* strains except CF2-7F. On the other hand, the addition of K^+^ reduced *α*-glucosidase of MF14-C to reach 13.71 ± 1.70 Glu U/mL. The growth of *L. reuteri* in the presence of K^+^ led to a significant (*p* < 0.05) decrease in *α*-glucosidase activity in all strains. The addition of Fe^2+^ also reduced *α*-glucosidase of MF14-C, 25.90 ± 2.72 Glu U/mL. Fe^2+^ decreased *α*-glucosidase activity of all *L. reuteri* strains except DSM20016 and MM2-3. Ca^2+^ enhanced *α*-glucosidase activity of DSM20016, MM7, and MM2-3 and decreased the activity of MF14-C, CF2-7F, and SD2112. Na^+^ enhanced *α*-glucosidase activity for all *L. reuteri* strains except MF14-C which was not affected.

**Table 2:** **Effect of metal ions on*α*-glucosidase activity (Glu U/mL) produced by*L. reuteri***

	***α***-Glucosidase activity (Glu U/mL)*						
***L. reuteri***	Control	Fe^2+^	Ca^2+^	Na^+^	K^+^	Mg^2+^	Mn^2+^
MF14-C	40.06	25.90	26.32	38.38	13.71	61.74	58.31
	±2.80^bA^	±2.72^cA^	±3.50^cB^	±1.98^bB^	±1.70^dBC^	±3.09^aA^	±2.88^aA^
CF2-7F	34.38	20.48	25.20	47.59	10.52	46.30	32.83
	±1.36^bB^	±1.75^cB^	±1.83^cB^	±1.86^aA^	±1.30^dC^	±2.64^aB^	±2.91^bC^
DSM20016	31.80	30.20	41.69	46.14	26.22	50.64	55.52
	±2.01^dBC^	±2.27^dA^	±2.89^cA^	±2.66^bcA^	±1.09^dA^	±1.30^aB^	±4.68^aA^
SD2112	29.34	19.54	20.57	34.58	15.59	35.51	40.55
	±1.27^cC^	±3.89 ^dB^	±1.82^dC^	±2.32^abB^	±1.11^dB^	±2.74^abC^	±1.90^aB^
MM7	25.32	19.40	35.93	37.57	12.59	28.13	35.68
	±2.32^cdCD^	±1.75^deB^	±3.62^abA^	±4.39^aB^	±1.83^eBC^	±3.22^bcD^	±2.63^abC^
MM2-3	20.65	18.28	24.79	24.98	14.79	44.11	41.42
	±1.70^bcE^	±1.49^bcB^	±2.72^bB^	±2.30^bC^	±2.60^eB^	±3.20^aB^	±3.66^aB^

*Data points with different lower case letters in the same row are significantly (*p* < 0.05) different. Data points with different upper case letters in the same column are significantly (*p* < 0.05) different.

**Figure 3:**
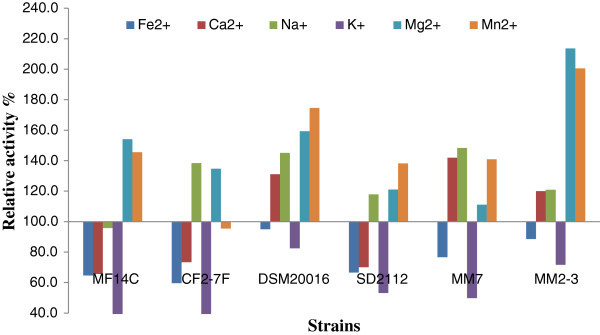
**Relative activity (%) of*α*-glucosidase produced by*L. reuteri*grown is SPM with added metal ions compared to the control group without metal ions.** The relative activity was calculated as the enzymatic activity in SPM with added metal ions divided by the enzymatic activity in SPM without metal ions then multiplied by 100.

Figure 3 shows the relative activity (%) of *α*-glucosidase produced by *L. reuteri* in the presence of metal ions. *α*-Glucosidase activity of MM2-3 growing in SPM with the addition of Mg^2+^ and Mn^2+^ was increase by 113.6% and 100.6% respectively. Mg^2+^ and Mn^2+^ also increased *α*-glucosidase activity of DSM20016 (by 59.2 and 74.6%) and MF14-C (by 54.1 and 45.6%) respectively. Mg^2+^ and Mn^2+^ were also found earlier to stimulate *α*-glucosidase activity of *L. acidophilus*(Li and Chan 1983). The addition of Na^+^ also enhanced *α*-glucosidase activity of the tested *L. reuteri* strains while showing less effect than Mg^2+^ and Mn^2+^. On the other hand, the growth of *L. reuteri* in SPM with added K^+^ led to a decrease in *α*-glucosidase ranged between 17.5 – 65.8%. Thus the effect of metal ions on *α*-glucosidase activity of *L. reuteri* is strain specific. These results come in agreement with previous studies. For example, *α*-glucosidase produced by *L. rhamnosus* R was inhibited by Hg^2+^, Mn^2+^, Cu^2+^, Fe^2+^ and Zn^2+^ and slightly activated by Li^+^, Na^+^, K^+^, Ca^2+^, Co^2+^, and Mg^2+^ (Pham et al. 2000). In addition, our results suggest the use of MF14-C and DSM20016 in the presence of Mg^2+^ and Mn^2+^ to produce enhanced levels of *α*-glucosidase.

### Induction of *β*-glucosidase by metal ions

In control group, *β*-glucosidase activity of *L. reuteri* ranged between 6.94 ± 1.29 and 17.82 ± 1.45 Glu U/mL for MF14-C and MM2-3 respectively (Table 3). The addition of K^+^ increased *β*-glucosidase produced by *L. reuteri* MF14-C and MM2-3 to reach 13.71 ± 1.70 and 24.79 ± 2.60 Glu U/mL respectively. K^+^ also increased *β*-glucosidase *L. reuteri* DSM20016 to reach the highest activity among the tested strains, 26.22 ± 1.09 Glu U/mL. The addition of Ca^2+^ or Fe^2+^ to SPM significantly (*p* < 0.05) enhanced *β*-glucosidase activity in all tested strains. Mg^2+^ and Mn^2+^ also enhanced *β*-glucosidase activity in the tested strains but showed lower enhancement effect compared to Ca^2+^, Fe^2+^ or K^+^. The lowest *β*-glucosidase with metal ions was produced by *L. reuteri* MF14-C in the presence of Ca^2+^ (9.66 ± 2.17 Glu U/mL). Thus, the addition of metal ions to SPM could enhance *β*-glucosidase activity of *L. reuteri*.

**Table 3:** **Effect of metal ions on*β*-glucosidase activity (Glu U/mL) produced by*L. reuteri***

	***β***-Glucosidase activity (Glu U/mL)*						
***L. reuteri***	Control	Fe^2+^	Ca^2+^	Na^+^	K^+^	Mg^2+^	Mn^2+^
MF14-C	6.94	11.46	9.66	11.18	13.71	10.15	12.30
	±1.29^cC^	±1.66^abC^	±2.17^bC^	±1.60^abC^	±1.70^aBC^	±1.74^bD^	±1.29^aB^
CF2-7F	10.11	18.02	21.93	16.86	10.52	13.60	16.71
	±1.58^cB^	±2.21^aAB^	±2.05^aA^	±1.12^abB^	±1.30^cD^	±1.13^bC^	±1.93^abA^
DSM20016	12.04	21.05	22.45	18.24	26.22	20.57	16.87
	±1.05^dB^	±2.23^bA^	±1.70^bA^	±1.75^bcB^	±1.09^aA^	±1.28^bAB^	±1.08^cdA^
SD2112	7.59	17.21	17.44	10.46	15.59	17.50	13.28
	±1.20^cdC^	±1.94^aAB^	±1.90^aB^	±1.69^cC^	±1.11^abB^	±1.72^aBC^	±1.50^bcB^
MM7	7.92	19.32	17.57	12.37	12.59	14.28	9.82
	±0.88^cC^	±2.94^aAB^	±1.70^aB^	±1.75^bC^	±1.83^bCD^	±1.60^abC^	±1.68^cC^
MM2-3	17.82	23.60	22.70	23.48	24.79	22.57	17.52
	±1.45^bA^	±2.27^aA^	±3.27^aA^	±3.21^aA^	±2.60^aA^	±3.24^aA^	±2.06^bA^

*Data points with different lower case letters in the same row are significantly (*p* < 0.05) different. Data points with different upper case letters in the same column are significantly (*p* < 0.05) different.

**Figure 4:**
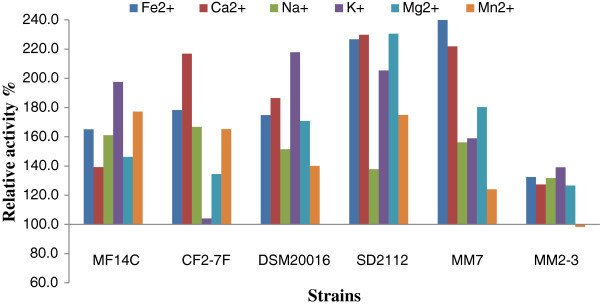
**Relative activity (%) of*β*-glucosidase produced by*L. reuteri*grown in SPM with added metal ions compared to the control group without metal ions.** The relative activity was calculated as the enzymatic activity in SPM with added metal ions divided by the enzymatic activity in SPM without metal ions then multiplied by 100.

The growth of *L. reuteri* in the presence of metal ions led to relative change in *β*-glucosidase ranged between -1.7 to 143.9% (Figure 4). The addition of Fe^2+^ enhanced *β*-glucosidase activity produced by *L. reuteri* MM7 and *L. reuteri* SD2112 by 143.9% and 126.7% respectively. Ca^2+^ enhanced *β*-glucosidase activity produced by *L. reuteri* MM7 and *L. reuteri* SD2112 by 121.8% and 129.8% respectively. The addition of K^+^ enhanced *β*-glucosidase produced by *L. reuteri* CF2-7F and *L. reuteri* SD2112 by 4.1% and 117.8% respectively. The effect of metal ions on *β*-glucosidase activity of *L. reuteri* MM2-3 was relatively low compared to other strains. Thus, the effect of metal ions on *β*-glucosidase activity produced by *L. reuteri* is strain dependent. Previous studies also showed that the effect of metal ions on *β*-glucosidase activity varied with the bacterial strain and type of metal ions (Pham et al. 2000). The effect of metal ions on *β*-glucosidase may be explained by that the bacterial sources of *β*-glucosidase have the highest activity and the most tolerance to inhibitors such as metal ions compare to other sources (Jeng et al. 2011). The growth of *L. reuteri* DSM20016 or MM2-3 in the presence of K^+^ could be used to produce high quantity of *β*-glucosidase. Thus, our results suggest the addition of Ca^2+^ and Fe^+2^ to produce enhanced levels of *β*-glucosidase.

*β*-Glucosidase activity of *L. reuteri* was determined in the disrupted cells. However, *β*-glucosidase was also tested in the supernatant after removal of the cells but only trace of enzyme activity was detected (data not shown). Thus, *β*-glucosidase produced by the tested *L. reuteri* strains is mainly cell-associated enzyme. *β*-Glucosidase was also reported to be a cell-associated enzyme in *L. acidophilus* (Mahajan et al. 2010) and *L. rhamnosus* R (Pham et al. 2000). The absence of *β*-glucosidase in the supernatant may suggest that most of extracted enzyme could be inactivated when separated from the cells.

### Induction of acid phosphatase by metal ions

**Figure 5:**
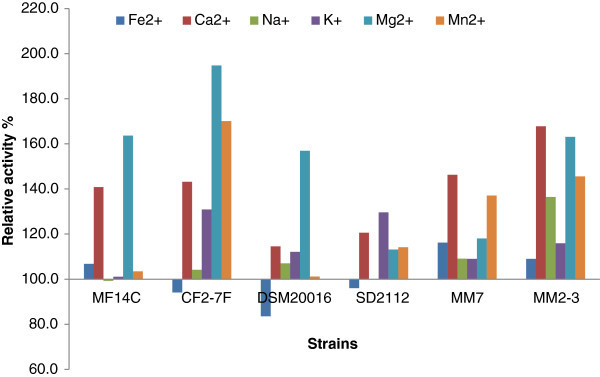
**Relative activity (%) of acid phosphatase produced by*L. reuteri*grown in SPM with added metal ions compared to the control group without metal ions.** The relative activity was calculated as the enzymatic activity in SPM with added metal ion divided by the enzymatic activity in SPM without metal ions then multiplied by 100.

Acid phosphatase in control group ranged between 8.73 ± 1.11 and 20.56 ± 0.74 Ph U/mL (Table 4). The addition of Mg^2+^ enhanced acid phosphatase activity of *L. reuteri* DSM20016 to reach the highest activity among tested strains, 29.33 ± 2.36 Ph U/mL. Mg^2+^ caused significant (*p* < 0.05) increase in acid phosphatase activity in all tested strains. Acid phosphatase produced by *L. reuteri* SD2112 was significantly (*p* < 0.05) increased in the presence of K^+^ or Ca^2+^ to reach 26.64 ± 1.39 and 24.78 ± 0.91 Ph U/mL respectively. The addition of Mn^2+^ increased acid phosphatase activity of *L. reuteri* CF2-7F by 10.1 units to reach 24.29 ± 2.54 Ph U/mL. Figure 5 shows the relative effect of metal ions on acid phosphatase. Acid phosphatase produced by *L. reuteri* CF2-7F was increased by 94.7% and 70.1% in the presence of Mg^2+^ and Mn^2+^ respectively. The addition of Ca^2+^ led to enhance acid phosphatase activity in *L. reuteri* MM2-3 by 67.8%. Fe^2+^ and Na^+^ caused only slight effect (decrease or increase) on acid phosphatase. For example Fe^2+^ decreased acid phosphatase activity of *L. reuteri* DSM20016 by 16.5%.

**Table 4:** **Effect of metal ions on acid phosphatase activity (Ph U/mL) produced by*L. reuteri***

	Acid phosphatase activity (Ph U/mL)*						
***L. reuteri***	Control	Fe^2+^	Ca^2+^	Na^+^	K^+^	Mg^2+^	Mn^2+^
MF14-C	13.59	14.51	19.14	13.49	13.74	22.24	14.06
	±1.51^bB^	±1.24^bB^	±1.51^aB^	±0.98^bB^	±0.74^bC^	±2.49^aB^	±0.90^bC^
CF2-7F	14.28	13.44	20.45	14.88	18.69	27.81	24.29
	±1.2^cB^	±1.11^cB^	±2.16^abB^	±1.57^cB^	±1.16^bB^	±2.43^aAB^	±2.54^aA^
DSM20016	18.69	15.61	21.41	20.00	20.96	29.33	18.91
	±1.15^bcA^	±2.40^cB^	±2.01^bB^	±1.27^bA^	±2.19^bB^	±2.36^aA^	±1.95^bcB^
SD2112	20.55	19.73	24.78	20.50	26.64	23.25	23.46
	±0.74^bA^	±0.36^bA^	±0.91^aA^	±0.95^bA^	±1.39^aA^	±2.38^aB^	±2.07^aA^
MM7	12.60	14.65	18.43	13.75	13.74	14.88	17.27
	±1.63^cB^	±0.98^abB^	±2.66^aB^	±2.37^bcB^	±1.20^bcC^	±1.31^abC^	±2.53^aBC^
MM2-3	8.73	9.52	14.65	11.91	10.12	14.24	12.71
	±1.11^cC^	±1.14^bcC^	±1.21^aC^	±1.41^abBC^	±1.31^bcD^	±1.49^aC^	±1.37^abCD^

*Data points with different lower case letters in the same row are significantly (*p* < 0.05) different. Data points with different upper case letters in the same column are significantly (*p* < 0.05) different.

The relative activity data suggested that the addition of Mg^2+^, Ca^2+^, or Mn^2+^ may lead to high increase in acid phosphatase. Mn^2+^ was reported to stimulate phosphatase activity which was explained by the fact that many protein phosphatases contain Mn^2+^ (Pallen and Wang 1985). However, the effect of metal ions on acid phosphatase produced by *L. reuteri* was found to be a strain dependent. Previous studies also showed that the effect of metal ions on acid phosphatase activity varied with bacterial strain (Aqel 2012; Palacios et al. 2005).

### Induction of phytase by metal ions

**Figure 6:**
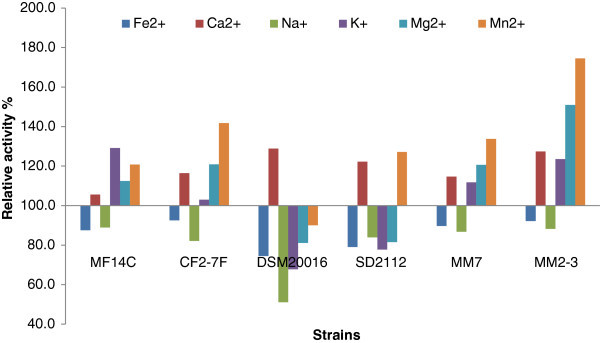
**Relative activity (%) of phytase produced by*L. reuteri*grown in SPM with added metal ions compared to the control group without metal ions.** The relative activity was calculated as the enzymatic activity in SPM with added metal ion divided by the enzymatic activity in SPM without metal ions then multiplied by 100.

Phytase activity in control group ranged between 0.51 ± 0.04 and 0.90 ± 0.05 Ph U/mL (Table 5). The growth of *L. reuteri* DSM20016 in the presence of Ca^2+^ led to the highest phytase activity, 1.16 ± 0.20 Ph U/mL. Phytase activity of *L. reuteri* SD2112 reached 1.03 ± 0.06 Ph U/mL in the presence of Mn^2+^. The addition of Ca^2+^ or Mn^2+^ to SPM enhanced phytase activity in all tested strains. On the other hand, phytase activity of *L. reuteri* DSM20016 was significantly (*p* < 0.05) decreased in the presence of Na^+^ to reach 0.46 ± 0.15 Ph U/mL. Phytase produced by *L. reuteri* DSM20016 was also reduced by Fe^2+^, K^+^, and Mg^2+^. Figure 6 shows the relative activity of *L. reuteri* in the presence of metal ions. Phytase produced by MM2-3 was increased by 74.5% in the presence of Mn^2+^. The addition Mg^2+^ enhanced phytase activity of MM2-3 by 51.0% and caused slight increase or decrease in the other strains. The addition of K^+^ enhanced phytase activity of MF14-C and MM2-3 and decreased phytase activity of DSM20016 and SD2112. Thus, our results suggested the addition of Mg^2+^, Mn^2+^, and Ca^2+^ to the culture media of *L. reuteri* to enhance the production of phytase. In addition, the effect of metal ions on phytase activity of *L. reuteri* was found to be a strain dependent.

**Table 5:** **Effect of metal ions on phytase activity (Ph U/mL) produced by*L. reuteri***

	Phytase activity (Ph U/mL)*						
***L. reuteri***	Control	Fe^2+^	Ca^2+^	Na^+^	K^+^	Mg^2+^	Mn^2+^
MF14-C	0.72	0.63	0.76	0.64	0.93	0.81	0.87
	±0.08^bcBC^	±0.06^cA^	±0.11^abBC^	±0.05^cAB^	±0.08^aA^	±0.05^abA^	±0.05^abB^
CF2-7F	0.67	0.62	0.78	0.55	0.69	0.81	0.95
	±0.05^bcC^	±0.07^bcA^	±0.08^bB^	±0.04^cB^	±0.05^bBC^	±0.06^bA^	±0.06^aA^
DSM20016	0.90	0.67	1.16	0.46	0.61	0.73	0.81
	±0.05^abA^	±0.04^cA^	±0.20^aA^	±0.15^cBC^	±0.23^bcBC^	±0.19^bAB^	±0.17^abA^
SD2112	0.81	0.64	0.99	0.68	0.63	0.66	1.03
	±0.06^bAB^	±0.05^cA^	±0.06^aA^	±0.03^bcA^	±0.04^cBC^	±0.08^bcB^	±0.06^aA^
MM7	0.68	0.61	0.78	0.59	0.76	0.82	0.91
	±0.04^bcC^	±0.06^cA^	±0.06^abB^	±0.09^cB^	±0.06^abB^	±0.04^aA^	±0.14^aAB^
MM2-3	0.51	0.47	0.65	0.45	0.63	0.77	0.89
	±0.04^cD^	±0.04^cB^	±0.04^bC^	±0.03^cC^	±0.11^bBC^	±0.05^bAB^	±0.03^aB^

*Data points with different lower case letters in the same row are significantly (*p* < 0.05) different. Data points with different upper case letters in the same column are significantly (*p* < 0.05) different.

The effect of metal ions on phytase activity of *Lactobacillus* was also investigated in previous studies. The addition of Ca^2+^ was previously reported to enhance phytase activity of *Lactobacillus* (Tang et al. 2010) and the addition of Fe^2+^ strongly inhibited phytase activity of *L. sanfranciscensis* (De Angelis et al. 2003). On the other hand, phytase activity of *L. reuteri* was found low compared to other tested enzymes. *Lactobacillus* strains had higher activity against *ρ*-nitrophenyl phosphate than phytate (Palacios et al. 2005). In addition, phytase does not seem to be common in *Lactobacillus* strains and phytase activity of *Lactobacillus* is generally low compared to other bacterial genera (De Angelis et al. 2003; Palacios et al. 2005). However, phytase and acid phosphatase are particular subgroups of phosphatases, whereas phytase exhibits a preference for phytate. The specificity of both acid phosphatase and phytase can partially overlap since acid phosphatase also shows phytase activity (Simon and Igbasan 2002). Thus, both acid phosphatase and phytase can be useful in the degradation of phytate.

## Conclusion

We studied the growth and enzymatic activity of *L. reuteri* in SPM. Our results demonstrate that the enzymatic activity of *L. reuteri* is strain dependent. α-Glucosidase activity of *L. reuteri* MFI4-C and DSM20016 was enhanced by Mg^2+^ and Mn^2+^. The addition of Ca^2+^, Fe^+2^, or K^+^ can enhance *β*-glucosidase activity of *L. reuteri* SD2112, DSM20016, and MM7. Acid phosphatase and phytase produced by MM2-3, CF2-7F, or MM7 could be increased by the addition of Mg^2+^, Ca^2+^, and Mn^2+^. Thus, to maximize the production of a target enzyme, it is required to select a combination of specific strain and specific metal ion. Nevertheless, Mn^2+^ and Mg^2+^ could be added to the culture media of *L. reuteri* to enhance the growth and enzymatic activity. Our results also revealed that more attention should be given to *L. reuteri* DSM20016 as high enzymatic activity is associated with this strain. Further studies need to done to investigate the optimum concentrations and possible combinations of metal ions that could be used to maximize the enzymatic activity of *L. reuteri*.
